# Therapeutic mechanism and clinical application of Chinese herbal medicine against diabetic kidney disease

**DOI:** 10.3389/fphar.2022.1055296

**Published:** 2022-11-03

**Authors:** Dan-Qian Chen, Jun Wu, Ping Li

**Affiliations:** ^1^ Department of Emergency, China-Japan Friendship Hospital, Beijing, China; ^2^ Shandong College of Traditional Chinese Medicine, Yantai, Shandong, China; ^3^ Beijing Key Lab for Immune-Mediated Inflammatory Diseases, Institute of Clinical Medical Sciences, China-Japan Friendship Hospital, Beijing, China

**Keywords:** diabetic kidney disease, Chinese herbal medicine, therapeutic mechanism, clinical application, metabolism regulation

## Abstract

Diabetic kidney disease (DKD) is the major complications of type 1 and 2 diabetes, and is the predominant cause of chronic kidney disease and end-stage renal disease. The treatment of DKD normally consists of controlling blood glucose and improving kidney function. The blockade of renin-angiotensin-aldosterone system and the inhibition of sodium glucose cotransporter 2 (SGLT2) have become the first-line therapy of DKD, but such treatments have been difficult to effectively block continuous kidney function decline, eventually resulting in kidney failure and cardiovascular comorbidities. The complex mechanism of DKD highlights the importance of multiple therapeutic targets in treatment. Chinese herbal medicine (active compound, extract and formula) synergistically improves metabolism regulation, suppresses oxidative stress and inflammation, inhibits mitochondrial dysfunction, and regulates gut microbiota and related metabolism *via* modulating GLP-receptor, SGLT2, Sirt1/AMPK, AGE/RAGE, NF-κB, Nrf2, NLRP3, PGC-1α, and PINK1/Parkin pathways. Clinical trials prove the reliable evidences for Chinese herbal medicine against DKD, but more efforts are still needed to ensure the efficacy and safety of Chinese herbal medicine. Additionally, the ideal combined therapy of Chinese herbal medicine and conventional medicine normally yields more favorable benefits on DKD treatment, laying the foundation for novel strategies to treat DKD.

## Introduction

Diabetic kidney disease (DKD), also called diabetic nephropathy (DN), is a microvascular complication of diabetes mellitus (DM) and characterized by microalbuminuria, declined glomerular filtration rate (GFR), and high risk of cardiovascular disease and stroke. DKD results in high morbidity and mortality worldwide (Koye et al., 2018). Clinically, DKD is defined as the presence of persistently increased urinary albumin (>300 mg/day) or urinary albumin-to-creatinine ratio (UACR>30 mg/g), accompanied by declined kidney function, eventually to end-stage renal disease (ESRD). According to pathological changes, DKD involves thickening of glomerular basement membrane, mesangial expansion, nodular sclerosis, and diabetic glomerulosclerosis (Thomas et al., 2015; Anders et al., 2018). DKD is the major complications of type 1 and 2 diabetes, and is the predominant cause of CKD that accounts for almost 50% of ESRD cases (Barrera-Chimal and Jaisser, 2020). Once DKD enters the dialysis stage, the economic burden of the patient and society greatly increases (Koye et al., 2018).

DKD treatment in early stage mainly focuses on the prevention of DM by the management of diet and lifestyle and glucose control. Once microalbuminuria occurred, the treatment needs to pay additional attention on alleviating and delaying albuminuria. Treatment targets hypertension, blood fat and uric acid also exhibit beneficial effect. The commonly used drugs are listed in Table 1. Although metformin, angiotensin-converting enzyme inhibitors (ACEIs), angiotensin receptor blockers (ARBs), glucagon-like peptide-1 (GLP-1) analogues and dipeptidyl peptidase-4 (DPP-4) inhibitors delay kidney function decline, the application of these drugs are limited. Recently, sodium glucose cotransporter 2 (SGLT2) inhibitor, as a novel type of hypoglycemic drugs, attaches a lot attention due to obvious advantages. SGLT2 excretes sugar directly through the kidneys, and only works when blood sugar exceeds the renal glucose threshold (Alicic et al., 2018). Functionally, SGLT2 inhibitors inhibit renal glucose reabsorption in the early proximal tubule, thereby lowering urinary glucose excretion and decreasing the glucose burden. In the diabetic nephron, compensatory upregulation and overexpression of the activity of SGLT2 glucose and sodium reabsorption in the proximal convoluted tubule results in decreased delivery of solutes to the macula densa. In the diabetic nephron with SGLT inhibition, lowering SGLT2-driven sodium-coupled glucose transport in the proximal convoluted tubule normalizes solute delivery to the macula densa, resulting in increasing solute and water reabsorption. However, adverse effects are unavoidable due to its mechanism, such as urinary tract infection and diabetic ketoacidosis. Therefore, the development of novel drugs and alternative strategies are urgently needed.

**TABLE 1:** The commonly used drugs in DKD treatment.

Treatment goal	Common drugs/cautions
Diet and lifestyle	Exercises, loss of weight, smoking cessation, protein intake, carbohydrate intake, fat intake, sodium intake and vitamin intake
Glucose control	Metformin, thiazolidinediones, GLP-1 analogues, DPP-4 inhibitors, and SGLT2 inhibitors
Hypertension control	ACEIs/ARBs, MRAs, CCBs, β-blocker, and diuretics
Albuminuria	ACEIs, ARBs, SGLT2 inhibitors, MRAs, and calcitriol impurities D
Blood lipid regulation	Statins and fibrates
Uric acid control	Diet control, allopurinol, and febuxostat

ARBs, angiotensin receptor blockers; ACEIs, angiotensin-converting enzyme inhibitors; CCBs, calcium channel blockers; DPP-4, dipeptidyl peptidase-4; GLP-1, glucagon-like peptide-1; MRAs, mineralocorticoid receptor antagonists; SGLT2, sodium/glucose cotransporter 2.

## Overview of the pathophysiological mechanism and therapeutic target of diabetic kidney disease

### Renin-angiotensin-aldosterone system

**FIGURE 1:**
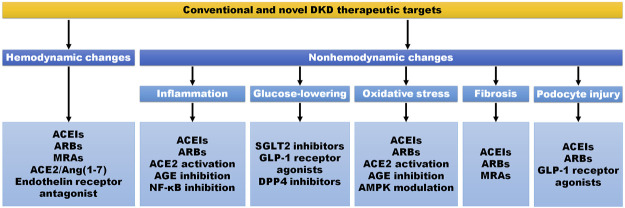
Conventional and novel DKD therapeutic targets and strategies. The therapeutic strategy of DKD includes hemodynamic and nonhemodynamic aspects. The improvement of hemodynamic changes, the glucose-lowering therapy, and the suppression of inflammation, oxidative stress, fibrosis and podocyte injury are the main therapeutic targets. ACE2, angiotensin-converting enzyme 2; ACEIs, angiotensin-converting enzyme inhibitors; AGE, advanced glycation end-product; AMPK, adenosine monophosphate-activated protein kinase; ARBs, angiotensin receptor blockers; DPP-4, dipeptidyl peptidase-4; GLP-1, glucagon-like peptide-1; MRAs, mineralocorticoid receptor antagonists; NF-κB, nuclear factor-kappa B; SGLT2, sodium glucose cotransporter 2.

According to the characteristics of DKD, its pathophysiological mechanisms include hemodynamic and nonhemodynamic mechanisms (Figure 1). In the early phase of DKD, intraglomerular hypertension and single-nephron hyperfiltration are responsible for renal injury (Tonneijck et al., 2017). The improvement of preglomerular (afferent) and postglomerular (efferent) arteriolar tone has therefore exhibited beneficial effects on DKD treatment. The renin-angiotensin-aldosterone system (RAAS) controls water and salt metabolisms, but during DKD process the overactivation of RAAS facilitates efferent constriction and intraglomerular hypertension to promotes disease progression (Warren et al., 2019). The inhibition of RAAS, such as ACEIs and ARBs, has been used as the first-line therapy for DKD treatment by maintaining arteriolar tone balance and decreasing albuminuria (Chen et al., 2018a). However, dual RAAS blockade exerts side effects for DKD treatment indicating the harm of efferent arteriole (Parving et al., 2012; Fried et al., 2013). The combined therapy of mineralocorticoid receptor antagonists (MRAs) with ACEIs or ARBs significantly decreases albuminuria and protects glomerular structure (Zhou et al., 2016; Barrera-Chimal et al., 2019; Barrera-Chimal et al., 2022). Notably, finerenone, a kind of MRAs, has been attracted a lot of attention for DKD treatment. Compared with placebo, finerenone treatment decreases albuminuria and lowers the risks of DKD progression and cardiovascular events in patients with CKD and type 2 diabetes (Bakris et al., 2020; Filippatos et al., 2021; Pitt et al., 2021). Additionally, angiotensin-converting enzyme 2 (ACE2)/Ang(1-7) axis exhibits protection on DKD treatment in animal studies (Chou et al., 2013; Liu et al., 2020a), indicating a promising therapeutic target against DKD. Additionally, RAAS also participates in DKD progression *via* nonhemodynamic mechanisms. The upregulation of Ang II contributes to DKD progression by activating proinflammatory and profibrotic effects (Yang et al., 2016b; Chen L. et al., 2017), while recombinant ACE2 attenuates DKD progression by suppressing oxidative stress, fibrosis, and mesangial cell proliferation (Malek et al., 2021).

### Endothelin receptor

The endothelin system accounts for sodium and water metabolism. Endothelin B receptor is responsible for the natriuresis and vasodilatation at the proximal tubule, whereas endothelin A receptor activation is involved in sodium retention and vasoconstriction (Stuart et al., 2013). Endothelin receptor antagonists reduce albuminuria and prevent renal function decline by dilating the efferent arteriole. According to both short-term and long-term clinical trials, treatment with atrasentan, the selective endothelin A receptor antagonist, significantly reduces albuminuria without inducing obvious sodium retention (Kohan et al., 2015), and decreases the risk of renal events in patients with diabetes and CKD (Heerspink et al., 2019).

### Sodium glucose cotransporter 2

For glucose-lowering therapies, SGLT2 inhibition and GLP-1 receptor agonists are the common choice for DKD treatment. The uptake and consumption of circulating glucose, the release of glucose by gluconeogenesis, and the reabsorption of glucose from glomerular filtrate are the main ways to maintain glucose homeostasis in the kidney (Alicic et al., 2018). SGLT2 controls tubular glucose reabsorption, and SGLT2 inhibition exhibits strong renal protection against DKD. SGLT2 inhibition improves solute delivery to the macula densa and reactivates tubuloglomerular feedback by reducing sodium and chloride reabsorption in the proximal tubule, which facilitates the reversal of afferent vasodilation and the normalization of glomerular hemodynamics. Several large clinical trials have proved the effective protection of empagliflozin and canagliflozin in patients with type 2 diabetes and CKD (Yale et al., 2013; Yale et al., 2014; Wanner et al., 2016; Cherney et al., 2017). Beyond the hemodynamic effect, reduced glucose uptake through the proximal tubular cells by SGLT2 inhibition alleviates DKD by inhibiting hyperglycemia-related tubulointerstitial injury (Anders et al., 2018; Kalantar-Zadeh et al., 2021).

### Glucagon-like peptide-1 and dipeptidyl peptidase-4

GLP-1 is secreted after food ingestion and reduces postprandial glucose levels by promoting insulin secretion, inhibiting glucagon release, delaying gastric emptying, and decreasing hepatic glucose production (Müller et al., 2019). The deletion of GLP-1 in animal model results in reduced albuminuria and mesangial expansion (Fujita et al., 2014). GLP-1 is degraded by the enzyme DPP-4 in a short time indicating that DPP-4 inhibition is suitable for clinical application rather than GLP-1 (Müller et al., 2019). Two type compounds GLP-1 receptor agonists and DPP-4 inhibitors are therefor used to treat DKD. Large clinical trials have confirmed the beneficial effects of GLP-1 receptor agonists on cardiovascular, mortality, and kidney outcomes in patients with type 2 diabetes, including lixisenatide, liraglutide, semaglutide, exenatide, albiglutide, dulaglutide, and oral semaglutide (Kristensen et al., 2019; Yamada et al., 2021). GLP-1 receptor agonist, liraglutide, also coordinates lipogenic and lipolytic signals and protects renal mitochondria function against renal injury by regulating sirtuin 1 (Sirt1)/AMP-activated kinase protein (AMPK)/peroxlsome proliferator-activated receptor-γ coactlvator-1α (PGC-1α) pathways (Wang et al., 2018). The possible mechanisms of DPP-4 inhibitors for treatment of DKD is a proximally acting natriuresis that increases sodium excretion and triggers tubuloglomerular feedback (Crajoinas et al., 2011). Large clinical trials indicate that DPP-4 inhibitors, dulaglutide and albiglutide, significantly decrease the frequency of major cardiovascular events in patients with type 2 diabetes and CKD (Hernandez et al., 2018; Gerstein et al., 2019). In addition to hemodynamic mechanism, DPP-4 inhibitors reduce albuminuria, alleviate glomerular sclerosis, and suppress oxidative stress and inflammation (Cappetta et al., 2019; Marques et al., 2019).

## Overview of Chinese herbal medicine in diabetic kidney disease treatment

A large number of studies have shown that Chinese herbal medicine has exhibited favorable efficacy on DKD treatment in clinics for decades, and has been the primary and additional treatment regimen. Chinese herbal medicine not only functions on abovementioned hemodynamic mechanisms also targets oxidative stress, glucose-lowering, inflammation, fibrosis, and podocyte injury to exert beneficial effects on DKD treatment (Chen et al., 2018b; Chen et al., 2019a), which attaches a lot attention. Notably, Chinese herbal medicine has been widely used to treat DKD clinically and yielded satisfactory results, which is recognized as a promisingly alternative therapy. Chinese herbal medicines are important sources for DKD treatment that prevents DKD and delays DKD progression by targeting multiple targets rather than single targets, including compounds, extracts, and Chinese herbal formulas. The present study aims to review the application of Chinese herbal medicines on DKD treatment in recent 3 years. We start from introducing DKD mechanisms and therapeutic targets, then summarize the advances on the therapeutic mechanisms and clinical application of Chinese herbal medicines on DKD treatment, and conclude by commenting on promising therapeutic candidates from Chinese herbal medicines to highlight the importance and capacity of Chinese herbal medicines on DKD treatment.

## Therapeutic mechanism of Chinese herbal medicine in diabetic kidney disease treatment

### Metabolism regulation

**FIGURE 2:**
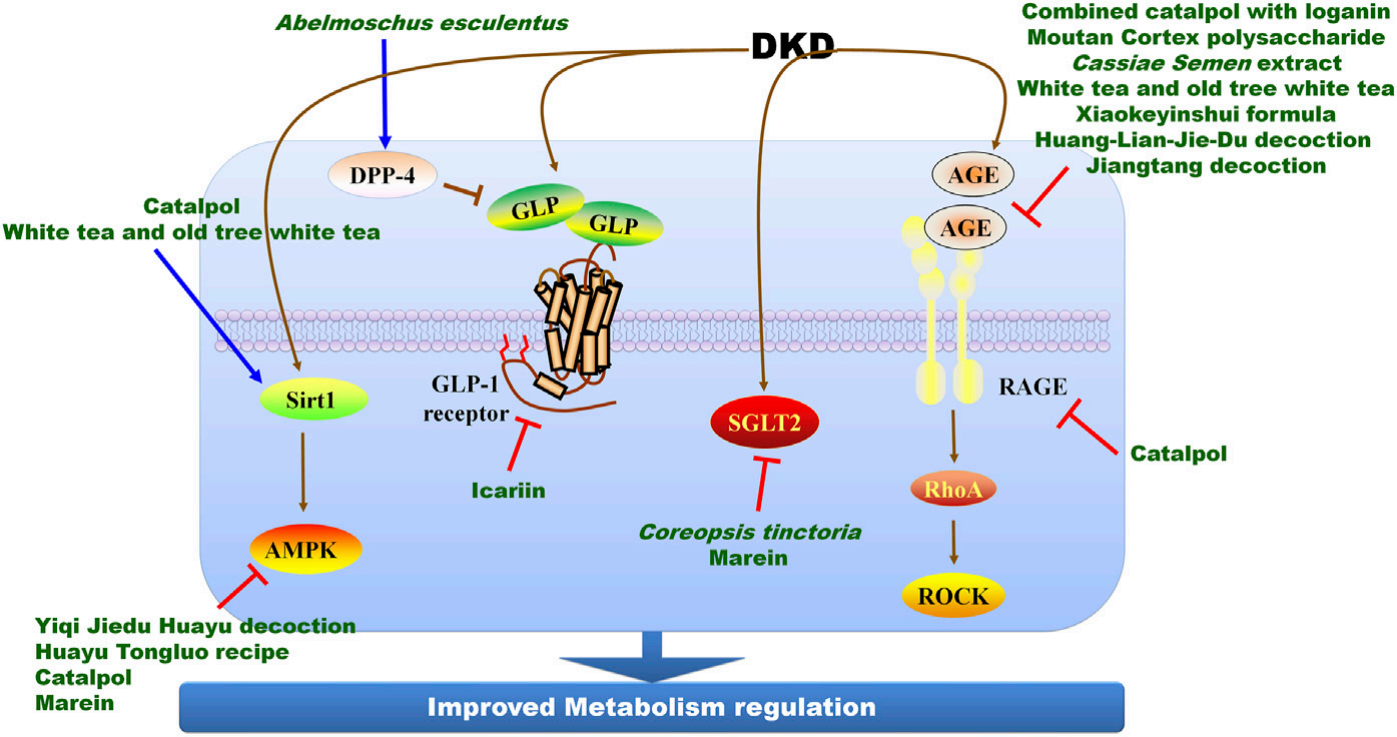
The molecular mechanisms and therapeutic targets of Chinese herbal medicine against DKD *via* improving metabolism regulation. AGE, advanced glycation end-product; AMPK, adenosine monophosphate-activated protein kinase; DPP-4, dipeptidyl peptidase-4; RAGE, receptors of AGE; GLP, glucagon-like peptide-1; ROCK, Rho-associated coiled-coil containing kinase; Sirt1, sirtuin 1.

Numerous Chinese herbal medicines have exhibited beneficial efficacy on DKD treatment in clinics. Here, we describe only some of the important findings for the sake of brevity, and introduce these important findings according to their potential therapeutic targets (Figure 2; Table 2). By targeting DPP-4 and GLP-1 receptor, *Abelmoschus esculentus* significantly inhibits oxidative stress and renal fibrosis to improve kidney function and alleviate diabetic renal damage in streptozocin (STZ)-induced model (Peng et al., 2019). Icariin, a flavonoid extracted from *Herba epimedii*, activates GLP-1 receptor to alleviate tubulointerstitial fibrosis in DKD rats (Jia et al., 2021). Beyond to GLP-1 receptor, icariin alleviates inflammation by inducing NOD-like receptor thermal protein domain associated protein 3 (NLRP3) inactivation and G protein-coupled estrogen receptor (GPER) activation *via* Kelch-1ike ECH-associated protein l (Keap1)-nuclear factor-erythroid-2-related factor 2 (Nrf2)/heme oxygenase-1 (HO-1) axis and inhibiting toll-like receptor 4 (TLR4)/nuclear factor-kappa B (NF-κB) signal pathway (Qiao et al., 2018; Wang et al., 2020; Qi et al., 2021; Ding et al., 2022), and prevents epithelial-mesenchymal transition (EMT) of tubular epithelial cells *via* modulating the miR-122-5p/forkhead box p2 axis against DKD (Zang et al., 2022).

**TABLE 2:** Therapeutic mechanisms and targets of Chinese herbal medicine in DKD treatment.

Chinese herbal medicine	Classification	Therapeutic mechanisms and targets	Reference
*Abelmoschus esculentus*	Herbal extract	Inhibiting DPP-4 and activating GLP-1 receptor	Peng et al. (2019)
Icariin	Active compound	Activating GLP-1 receptor, inactivating NLRP3, activating GPER *via* Keap1-Nrf2/HO-1 axis and inhibiting TLR4/NF-κB pathway	Qiao et al. (2018), Wang et al. (2020), Jia et al. (2021), Qi et al. (2021), Ding et al. (2022), and Zang et al. (2022)
Catalpol	Active compound	Modulating RAGE/RhoA/ROCK AMPK/Sirt1/NF-κB pathways	Chen Y. et al. (2019), Chen et al. (2020a), Chen et al. (2020b), and Shu et al. (2021)
Combined catalpol and loganin	Active compound	Inhibiting AGE/RAGE pathway	Chen et al. (2020b)
Novel polysaccharide	Active compound	Inhibiting AGEs and RAGEs levels	Lian et al. (2021)
*Cassiae Semen* extract	Herbal extract	Inhibiting AGEs and RAGEs levels	Wang et al. (2019)
White tea and old tree white tea	Herbal extract	Activating Sirt1/AMPK pathway	Xia et al. (2021)
Xiaokeyinshui formula	Formula	Inhibiting AGE/RAGE pathway	Zhou et al. (2020)
Huang-Lian-Jie-Du decoction	Formula	Inhibiting AGE/RAGE pathway	Tang et al. (2022)
Jiangtang decoction	Formula	Inhibiting AGE/RAGE pathway	Hong et al. (2017)
*Coreopsis tinctoria*	Herbal extract	Inhibiting SGLT2 and modulating NF-κB pathway	Yao et al. (2019) and Yu et al. (2019)
Marein and flavanomarein	Active compounds	Inhibiting SGLT2, and activating AMPK/ACC/PGC-1α pathway	Guo et al. (2020) and Zhang et al. (2020a)
Yiqi Jiedu Huayu decoction	Formula	Modulating AMPK pathway	Xuan et al. (2021)
Huayu Tongluo recipe	Formula	Modulating AMPK pathway	Li et al. (2021)
Curcumin	Active compounds	Activating Nrf2, inhibiting NF-κB, NADPH oxidase and PKCβII/p66Shc axis, and inhibiting NLRP3 inflammasome activity	Lu et al. (2017), Ghasemi et al. (2019), AlTamimi et al. (2021), and Xie et al. (2021)
*Fructus Arctii*	Herbal extract	Inhibiting ER stress signal transduction pathway	Zhang et al. (2019a)
Arctigenin	Active compounds	Enhancing PP2A activity	Zhong et al. (2019)
Baicalin	Active compounds	Inhibiting MAPK pathway	Ma et al. (2021)
Ellagic acid	Active compounds	Inhibiting MAPK pathway	Lin et al. (2021)
Astragaloside IV	Active compounds	Inhibiting NLRP3 inflammasome	Feng et al. (2021)
Yi Shen Pai Du Formula	Formula	Activating Nrf2 pathway	Zhang et al. (2021a)
Tangshen Formula	Formula	Modulating TXNIP-NLRP3-GSDMD axis	Li et al. (2020)
Berberine	Active compounds	Activating AMPK pathway to elevate PGC-1α, inhibiting Drp1-mediated mitochondrial fission and dysfunction, and stimulating the positive feedback loop of C/EBPβ/Gas5/miR-18a-5p	Qin et al. (2019), Qin et al. (2020), Rong et al. (2021), and Xu et al. (2021a)
*Rhodiola rosea* Salidroside	Herbal extract	Promoting mitochondrial DNA copy and electron transport chain proteins by enhancing Sirt1 and PGC-1α expression	Wang et al. (2013) and Xue et al. (2019)
Salidroside	Active compounds	Suppressing TXNIP-NLRP3 inflammasome pathway	Wu et al. (2016) and Wang et al. (2017)
Resveratrol	Active compounds	Suppressing mitochondrial oxidative stress	Zhang et al. (2019c)
4-O-methylhonokiol	Active compounds	Activating AMPK/PGC-1α/CPT1B pathway and activating Nrf2/SOD2 pathway	Ma et al. (2019)
Tangshen Formula and morroniside	Formula, Active compounds	Activating PGC-1α-LXR-ABCA1 pathway	Liu et al. (2018) and Gao et al. (2021a)
Astragaloside II	Active compounds	Upregulating PINK1 and Parkin	Su et al. (2021a)
Astragaloside IV	Active compounds	Inhibiting cytochrome c release and mitochondrial membrane potential	Xing et al. (2021) and Zang et al. (2021)
Combined Ginsenoside Rb1 and aldose therapy	Active compounds	Alleviating mitochondrial damage	He et al. (2022)
Quercetin	Active compounds	Modulating HIF-1α/miR-210/ISCU/FeS pathway	Xu et al. (2021b)
Andrographolide	Active compounds	Suppressing mitochondrial ROS-mediated NLRP3 inflammasome activation	Liu et al. (2021b)
Huangqi-Danshen decoction	Formula	Suppressing PINK1/Parkin-mediated mitophagy	Liu et al. (2020b)
Punicalagin	Active compounds	Reshaping gut microbial ecology, reversing gut barrier dysfunction, and reducing serum lipopolysaccharide and diamine oxidase levels	Hua et al. (2022)
Polysaccharide	Active compounds	Reconstructing gut microbiota, improving intestinal barrier function, ameliorating serum proinflammatory mediators, and upregulating short-chain fatty acid level	Zhang et al. (2022)
Qing-Re-Xiao-Zheng formula	Formula	Modulating gut microbiota-bile acid axis *via* farnesoid X receptor	Gao et al. (2021)
QiDiTangShen granules	Formula	Modulating gut microbiota-bile acid axis *via* farnesoid X receptor	Wei et al. (2021)
Shenyan Kangfu tablet	Formula	Improving intestinal microbiota *via* elevated *Firmicutes* and reduced *Bacteroidetes* abundance	Chen et al. (2021)
San-Huang-Yi-Shen capsule	Formula	The improvement of gut microbiota by modulating arginine biosynthesis, TCA cycle, tyrosine metabolism, and arginine and proline metabolism	Su et al. (2021b)
Tangshen Formula	Formula	Regulating gut microbiota to reducing lipopolysaccharide and indoxyl sulfate levels	Zhao et al. (2020)

ABCA1, ATP-binding cassette transporter A1; ACC, acetyl-CoA carboxylase; AGE, advanced glycation end-product; AMPK, adenosine monophosphate-activated protein kinase; CPT1B, carnitine palmitoyltransferase 1B; DPP-4, dipeptidyl peptidase-4; EBPβ, enhancer binding protein beta; ER, endoplasmic reticulum; Gas5, growth arrest-specific 5; GLP-1, glucagon-like peptide-1; GPER, G protein-coupled estrogen receptor; GSDMD, gasdermin D; HO-1, heme oxygenase-1; Keap1, kelch-1ike ECH- associated protein l; LXR, liver X receptor; MAPK, mitogen-activated protein kinase; NF-κB, nuclear factor-kappa B; NLRP3, NOD-like receptor thermal protein domain associated protein 3; PGC-1α, peroxlsome proliferator-activated receptor-γ coactlvator-1α; PINK1, PTEN induced putative kinase 1; PKCβII, Significant up-regulation of the protein kinase Cβ II; PP2A, protein phosphatase 2 A; RAGE, receptors of AGE; ROCK, Rho-associated coiled-coil containing kinase; ROS, reactive oxygen species; SGLT2, sodium glucose cotransporter 2; Sirt1, sirtuin 1; SOD2, superoxide dismutase 2; TLR4, toll-like receptor 4; TXNIP, thioredoxin-interacting protein.

Advanced glycation end-products (AGEs) are the non-enzymatic glycation products between the aldehyde group of saccharide and amino group of protein, lipid, or nucleic acid. AGEs result in the irreversible transformation of protein by glycation and facilitate DKD progression. The accumulation of AGEs and receptors of AGEs (RAGEs) accelerate glomerulus and tubule injury, and progressive proteinuria (Tang et al., 2021). AGE/AGE pathway is the pivotal therapeutic target of Chinese herbal medicine. Catalpol, an iridoid glycoside isolated from the root of *Rehmannia glutinosa*, significantly alleviates AGE/RAGE-induced endothelial dysfunction and inflammation in DKD mice and delays DKD progression *via* RAGE/RhoA/Rho-associated coiled-coil containing kinase (ROCK) pathway (Shu et al., 2021). The combined therapy of catalpol with loganin isolated from *Cornus officinalis* cooperatively prevents podocyte apoptosis by targeting AGE/RAGE pathway, and exerts stronger effects than used alone against DKD (Chen et al., 2020b). Catalpol also stabilizes podocyte cytoskeleton and enhances injured podocyte autophagy to prevent DKD by suppressing mammalian target of rapamycin activity and promoting transcription factor EB nuclear translocation, and inhibits oxidative stress and inflammation *via* AMPK/Sirt1/NF-κB pathway (Chen Y. et al., 2019; Chen et al., 2020a). The novel polysaccharide isolated from *Moutan Cortex* significantly reduces serum AGE and RAGE levels to prevent DKD progression in rat model (Lian et al., 2021). In addition to active compounds from Chinese herbal medicine, *Cassiae Semen* extract obviously controls glucose and lipid metabolism, and suppresses oxidative stress and inflammatory responses *via* regulating AGEs and RAGEs against DKD in STZ-induced rat model (Wang et al., 2019). White tea and old tree white tea ameliorate AGE accumulation in kidney of STZ-induced mouse model, and alleviate oxidative stress and inflammation *via* activating Sirt1/AMPK pathway (Xia et al., 2021). Additionally, Chinese herbal formula including Xiaokeyinshui formula, Huang-Lian-Jie-Du decoction and Jiangtang decoction exhibit renal protective effects in DKD animal models *via* regulating AGE/RAGE pathway (Hong et al., 2017; Zhou et al., 2020; Tang et al., 2022).

SGLT2 is an important therapeutic target of Chinese herbal medicine. *Coreopsis tinctoria* Nutt is widely used to treat high blood pressure and diarrhea, and SGLT2 is its potential therapeutic target. Its alcohol extract protects diabetic kidney injury in db/db mice by suppressing miR-192- and miR-200b-mediated phosphatase and tensin homolog deleted on chromosome ten (PTEN)/phosphoinositide 3-kinase (PI3K)/AKT pathway (Yu et al., 2019), while its ethyl acetate extract controls transforming growth factor-β1/Smads, AMPK, and NF-κB signaling pathways to delay DKD progression (Yao et al., 2019). Further study shows that marein and flavanomarein are the active compounds of *C. tinctoria* to prevent DKD progression. Marein directly inhibits SGLT2 expression and then activates AMPK/acetyl-CoA carboxylase (ACC)/PGC-1α pathway to correct hyperglycemia and dyslipidemia and diabetic kidney injury in db/db mice (Guo et al., 2020). Spleen tyrosine kinase are the potential therapeutic target of flavanomarein to ameliorate high glucose-induced extracellular matrix (ECM) against DKD (Zhang et al., 2020a). Additionally, Yiqi Jiedu Huayu decoction and Huayu Tongluo recipe suppress diabetic kidney injury in STZ-induced rat model *via* modulating AMPK pathway (Li et al., 2021; Xuan et al., 2021).

### The inhibition of oxidative stress and inflammation

**FIGURE 3:**
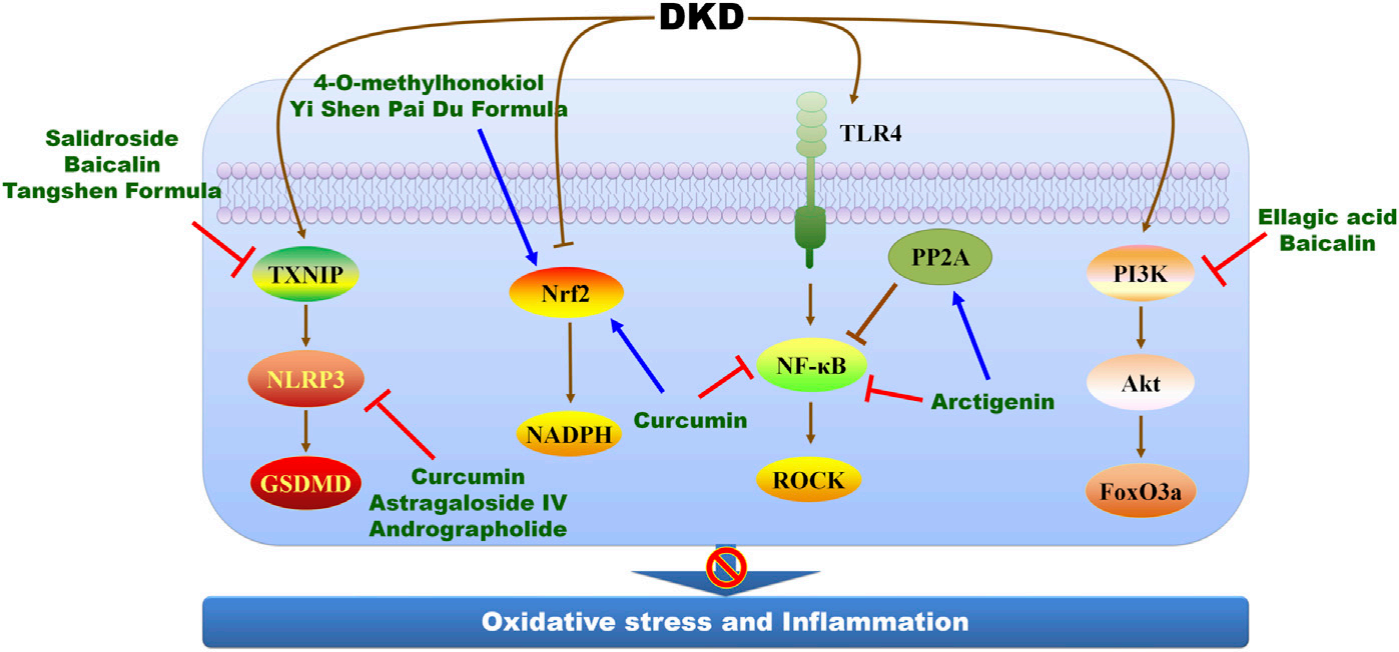
The molecular mechanisms and therapeutic targets of Chinese herbal medicine against DKD *via* suppressing oxidative stress and inflammation. FoxO3a, forkhead box transcription factor 3a; GSDMD, gasdermin D; NF-κB, nuclear factor-kappa B; NLRP3, NOD-like receptor thermal protein domain associated protein 3; Nrf2, nuclear factor-erythroid 2-related factor 2; PI3K, phosphoinositide 3-kinase; PP2A, protein phosphatase 2 A; ROCK, Rho-associated coiled-coil containing kinase; TLR4, toll-like receptor 4; TXNIP, thioredoxin-interacting protein.

Oxidative stress and inflammation drive the development of DKD, and several novel mediators are involved in such process, including tonicity-responsive enhancer-binding protein, apoptosis signal-regulating kinase 1, serine/threonine protein kinase 25, and receptor activator of NF-κB (Chen et al., 2017a; Chen et al., 2017b; Liles et al., 2018; Chen et al., 2019b; Cansby et al., 2020; Choi et al., 2020; Ke et al., 2021; Liu et al., 2022b). Additionally, superoxide dismutase (SOD), glutathione peroxidase, malondialdehyde are considered as biomarkers of oxidative stress to evaluate the effects of Chinese herbal medicine on DKD treatment (Zhou et al., 2022). Even mechanistic studies concerning DKD progress develop a lot, mechanistic studies concerning Chinese herbal medicine against DKD is relatively hysteretic, in that most of Chinese herbal medicine alleviates oxidative stress and inflammation in DKD by modulating NF-κB and Nrf2 pathways (Figure 3; Table 2). Curcumin significantly ameliorates albumin/protein urea and increased creatinine clearance in STZ-induced DKD rats, which involves the activation of Nrf2 and the inhibition of NF-κB, NADPH oxidase and significant up-regulation of the protein kinase Cβ II (PKCβII)/p66^Shc^ axis (AlTamimi et al., 2021). The inhibition of NLRP3 inflammasome activity and the downregulation of kidney injury molecule 1 and neutrophil gelatinase-associated lipocalin are also therapeutic targets of curcumin to suppress oxidative stress against DKD progression (Lu et al., 2017; Ghasemi et al., 2019). The combination of curcumin with antihyperglycemic agents exerts stronger effects against diabetic complications by maintaining Nrf2 pathway homeostasis (Xie et al., 2021). *Fructus Arctii* attenuates proteinuria in patients with diabetics, and arctigenin, a lignan extracted from *F. Arctii*, reduces proteinuria and podocyte injury in diabetes mouse models. Arctigenin enhances protein phosphatase 2 A (PP2A) activity to alleviate p65-mediated inflammation *in vivo*, and blocks endoplasmic reticulum (ER) stress signal transduction pathway to suppress apoptosis in high glucose-induced HK2 cells (Zhang et al., 2019a; Zhong et al., 2019). Specific deletion of PP2A in podocyte weakens the efficiency of arctigenin, indicating PP2A as the therapeutic target of arctigenin (Zhong et al., 2019).

Beyond to NF-κB and Nrf2 pathways, the suppression of mitogen-activated protein kinase (MAPK)-mediated inflammatory signaling pathway is therapeutic target of baicalin. Baicalin attenuates diabetic conditions, proteinuria, renal histopathological changes, and alleviates oxidative stress and inflammation in DKD animal model *via* Nrf2 and MAPK pathways (Ma et al., 2021). Ellagic acid alleviates high glucose-induced mesangial cell injury and inflammation in a concentration-dependent manner, and the underlying mechanisms involve in the activation of PI3K/Akt signaling pathway and the suppression of forkhead box transcription factor 3a (FoxO) 3a during DKD (Lin et al., 2021). Astragaloside IV alleviates podocyte injury and delays DKD progression in db/db mice *via* suppressing NLRP3 inflammasome-mediated inflammation (Feng et al., 2021). Yi Shen Pai Du Formula inhibits oxidative stress, inflammation, and EMT to delay DKD progression in db/db *via* activating Nrf2 pathway (Zhang et al., 2021a). Tangshen Formula, a Chinese formulation, exerts beneficial effects against DKD by modulating thioredoxin-interacting protein (TXNIP)-NLRP3-gasdermin D (GSDMD) axis-mediated pyroptosis in STZ-induced rat model and AGE-induced HK-2 cells (Li et al., 2020).

### The modulation of mitochondrial dysfunction

**FIGURE 4:**
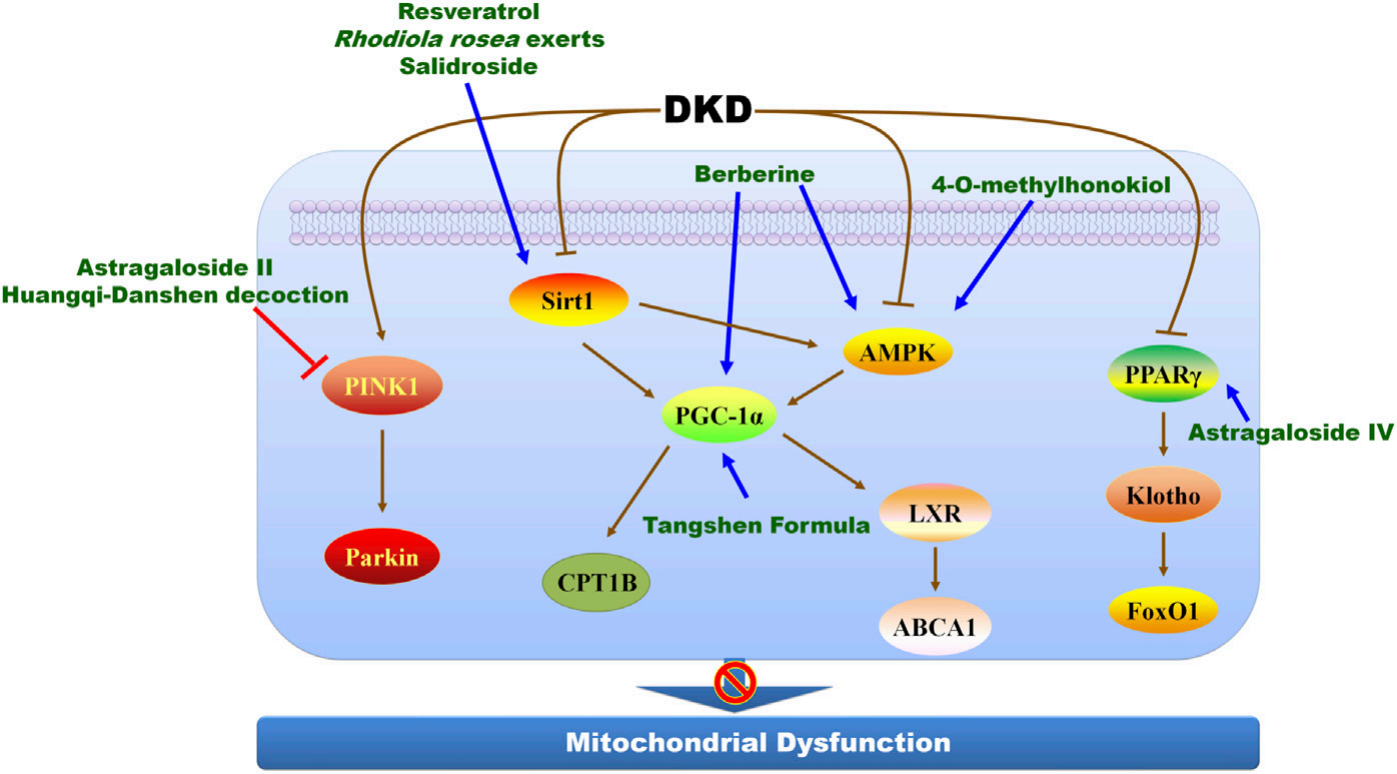
The molecular mechanisms and therapeutic targets of Chinese herbal medicine against DKD *via* alleviating mitochondrial dysfunction. ABCA1, ATP-binding cassette transporter A1; AMPK, adenosine monophosphate-activated protein kinase; CPT1B, carnitine palmitoyltransferase 1B; FoxO1, forkhead box transcription factor 1; LXR, liver X receptor; PGC-1α, peroxlsome proliferator-activated receptor-γ coactlvator-1α; PINK1, PTEN induced putative kinase 1; PPARγ, peroxisome proliferator-activated receptor gamma; Sirt1, sirtuin 1.

PGC-1α is a prominent modulator of mitochondrial biogenesis and an attractive therapeutic target in DKD treatment (Li and Susztak, 2016; Long et al., 2016; Trembinski et al., 2020), and several Chinese herbal medicines and their active compounds exert renoprotection against DKD by modulating PGC-1α (Figure 4; Table 2). Metabolomic research proves that the abnormality on mitochondrial fuel usage and mitochondrial dysfunction occurs in patients with DKD, and berberine, the main active compounds of *Rhizoma coptidis* and *Cortex phellodendri*, modulates PGC-1α to alleviate mitochondrial injury in direct and indirect pathways. Berberine facilitates mitochondrial energy homeostasis and fatty acid oxidation by directly activating PGC-1α signaling pathway and protects glomerular podocytes *via* inhibiting dynamin-related protein 1-mediated mitochondrial fission and dysfunction in db/db mice model and cultured podocytes (Qin et al., 2019; Qin et al., 2020). Berberine activates AMPK pathway to upregulate PGC-1α that reduces fatty acid oxidation, lipid deposition, and protects mitochondria to mitigate diabetic renal tubulointerstitial injury (Rong et al., 2021). Additionally, berberine modulates mitochondrial reactive oxygen species (ROS) generation by stimulating the positive feedback loop of CCAAT enhancer binding protein beta (C/EBPβ)/growth arrest-specific 5 (Gas5)/miR-18a-5p (Xu et al., 2021a). The ethanol extract of *Rhodiola rosea* exerts beneficial protection in STZ-induced model in the early nephropathy in type 2 diabetic rats (Wang et al., 2013). Further study identifies salidroside as a major active compound of *R. rosea* to treat DKD. Salidroside markedly improves renal structures and reverses the downregulation of nephrin and podocin in patients with DKD. Mechanistically, salidroside treatment promotes mitochondrial DNA copy and electron transport chain proteins by enhancing Sirt1 and PGC-1α expression in STZ-induced mice (Xue et al., 2019). Salidroside also suppresses oxidative stress and ECM accumulation by modulating TXNIP-NLRP3 inflammasome pathway (Wang et al., 2017), and reduces proteinuria by attenuating caveolin-1 phosphorylation and albumin transcytosis across glomerular endothelial cells (Wu et al., 2016). Resveratrol, a potent Sirt1 agonist, attenuates podocyte damage in diabetic mice by suppressing mitochondrial oxidative stress (Zhang et al., 2019c), while 4-O-methylhonokiol, isolated from *Magnolia stem bark*, protects against STZ-induced DKD by activating AMPK/PGC-1α/carnitine palmitoyltransferase 1B (CPT1B)-mediated fatty acid oxidation and Nrf2/SOD2-mediated anti-oxidative stress (Ma et al., 2019). Notably, Tangshen Formula and its active compounds morroniside enhances renal cholesterol efflux to ameliorates tubular epithelial injury in db/db mice by activating PGC-1α-liver X receptor (LXR)-ATP-binding cassette transporter A1 (ABCA1) pathway (Liu et al., 2018; Gao et al., 2021a), indicating a promising candidate for DKD treatment.

Other potential therapeutic targets are also proved, including PTEN induced putative kinase 1 (PINK1) and peroxisome proliferator-activated receptor (PPAR) (Figure 4; Table 2). Several studies show the renoprotective effects of astragaloside II and astragaloside IV, active compounds of *Astragalus membranes*, referring to the improvement of mitochondrial dysfunction. Astragaloside II and astragaloside IV obviously ameliorate albuminuria in DKD animal models and prevent podocyte injury from high glucose (Su et al., 2021a; Xing et al., 2021; Zang et al., 2021). Astragaloside II suppresses mitochondrial dysfunction of podocyte injury *via* upregulating PINK1 and Parkin, and astragaloside IV inhibits cytochrome c release and mitochondrial membrane potential to protect podocyte against DKD (Su et al., 2021a; Zang et al., 2021). Astragaloside II also mitigates podocyte apoptosis against DKD *via* suppressing transient receptor potential channel 6-mediated Ca2^+^ influx, and astragaloside IV activates PPARγ-Klotho-FoxO1 pathway to alleviate podocyte apoptosis (Xing et al., 2021; Zang et al., 2021). Ginsenoside Rb1 combines with aldose reductase to alleviate mitochondrial damage and podocyte apoptosis thereby delaying the progression of DKD (He et al., 2022). Quercetin, an active compound from *Panax notoginseng*, antagonizes glucose fluctuation-caused kidney injury by inhibiting aerobic glycolysis *via* hypoxia inducible factor-1 alpha (HIF-1α)/miR-210/ISCU/FeS pathway in glomerular mesangial cells (Xu et al., 2021b). Andrographolide isolated from *Andrographis paniculate* suppresses mitochondrial ROS-mediated NLRP3 inflammasome activation to ameliorate mitochondrial dysfunction during DKD (Liu et al., 2021b). Chinese herbal formula Huangqi-Danshen decoction delay DKD progress by suppressing PINK1/Parkin-mediated mitophagy (Liu et al., 2020b). Additionally, signal transducer and activator of transcription 3 is also a potential therapeutic target to modulate mitochondrial homeostasis through SDF-1α/CXCR4 pathway to ameliorate renal tubular injury in DKD (Zhang et al., 2020b).

### The regulation of gut microbiota and related metabolism

Emerging evidences have confirmed the relationship between the dysfunction of gut microbiota and related metabolism and the progression of DKD indicating the importance of kidney-gut axis (Winther et al., 2020; Yang et al., 2021). According to 16S rRNA sequencing and metabolomic results, the gut microbiota structure, phenylalanine and tryptophan metabolic pathways are significantly altered in patients with DKD (Zhang et al., 2021b). Phenyl sulfate is a gut microbiota-derived metabolite, and its level increases with the progression of diabetes in rat model. Phenyl sulfate obviously contributes to albuminuria and could be used as a biomarker for DKD (Kikuchi et al., 2019). Trimethylamine *N*-oxide (TMAO) is a gut microbiota-derived metabolite, and serum TMAO closely relates to and mediates impaired renal function (Winther et al., 2019). As an important component of innate immunity, mitochondrial antiviral signaling protein (MAVS) maintains intestinal integrity. DKD contributes to the impairment of MAVS signaling in the kidney and intestine thus leading to the disrupted homeostasis, indicating that maintaining intestinal homeostasis may functions a novel therapeutic approach for DKD treatment (Linh et al., 2022).

Notably, gut microbiota and related metabolism are the potential therapeutic target of Chinese herbal medicine against DKD (Table 2). Punicalagin isolated from *Punica granatum* reshapes gut microbial ecology, reverses gut barrier dysfunction, and reduces serum lipopolysaccharide and diamine oxidase levels to delay DKD progression (Hua et al., 2022). Functioning as a prebiotic, the polysaccharide extracted from *M. Cortex* reconstructs gut microbiota, improves intestinal barrier function, ameliorates serum proinflammatory mediators, and upregulates short-chain fatty acid level by controlling *Lactobacillus* and *Muribaculaceae_unclassified* abundance in gut of DKD rat model (Zhang et al., 2022). Qing-Re-Xiao-Zheng formula reverses gut dysbiosis and inhibits generation of gut-derived LPS, and suppresses DKD-related inflammation by reducing TLR4 and NF-κB expression in DKD mouse model (Gao et al., 2021b). QiDiTangShen granules exert good efficacy on alleviating proteinuria in DKD mice model, and the underlying mechanisms involves the modulation of gut microbiota-bile acid axis *via* farnesoid X receptor (Wei et al., 2021). Shenyan Kangfu tablet, a prescription of traditional Chinese medicine, attenuates stimulated blood glucose and glycosylated hemoglobin (HbA1c) levels and alleviates renal dysfunction and inflammation in db/db mice. The underlying mechanisms refer to suppressed renal inflammatory signaling cascades and improved intestinal microbiota *via* elevated *Firmicutes* and reduced *Bacteroidetes* abundance (Chen et al., 2021). San-Huang-Yi-Shen capsule exhibits beneficial effects against DKD in clinics. The mechanism involves the improvement of gut microbiota by modulating arginine biosynthesis, tricarboxylic acid (TCA) cycle, tyrosine metabolism, and arginine and proline metabolism (Su et al., 2021b). In addition to above-mentioned mechanisms, Tangshen Formula attenuates diabetic renal injury and inflammation by regulating gut microbiota to reducing lipopolysaccharide and indoxyl sulfate levels (Zhao et al., 2020).

## Clinical application of Chinese herbal medicine in diabetic kidney disease treatment

Even a lot of work engages to elucidate the underlying mechanisms of Chinese herbal medicine, the lack of high-quality evidences from clinical trials significantly hinders the application of Chinese herbal medicine worldwide. Here, we summarize randomized clinical trials (RCTs) of Chinese herbal medicine against DKD to highlight its beneficial efficacy, and found that Chinese herbal medicine can be used as the primary and additional treatment regimen for DKD in clinics.

Single use of Chinese herbal medicine shows beneficial efficacy on DKD treatment. A retrospective study reports the beneficial efficacy of a traditional Chinese medicine, Shenzhuo formula, on patients with DKD. The changes in estimated GFR (eGFR), creatinine clearance, serum creatinine, blood urea nitrogen, albuminuria, HbA1c, blood pressure, and lipid profile are observed. Compared with the baseline, serum creatinine significantly decreases, and estimated glomerular filtration rate (eGFR) and creatinine clearance increases after intervention at 1, 3, 6, 9, 12, and 18 months. Shenzhuo formula also reduces HbA1c, lipid levels and blood pressure (Tian et al., 2015). A multicenter, parallel-control, open-label, RCT investigates the effect of Zicuiyin decoction on DKD treatment, and the primary outcome is the change of eGFR. Zicuiyin decoction significantly increases eGFR and decreases serum creatinine to alleviate DKD *via* correcting gut microbiota dysbiosis (Liu et al., 2022a). Additionally, a single-blind, randomized, controlled preliminary study explores the efficacy of the acupressure at Sanyinjiao for DKD treatment, and the primary outcome measure is the UACR or logarithmic transformed UACR (log-UACR) changes. The difference in UACR and log-UACR before and after the study was higher in the Sanyinjiao group than in the sham groups, and the acupressure at Sanyinjiao for 8 weeks helps to decrease albuminuria in patients with early DKD indicated by eGFR and HbA1c (Chuang et al., 2020).

Combined therapy of Chinese herbal medicine and ARB/ACEI also exhibit favorable efficacy on DKD treatment. Huangkui capsule from traditional Chinese medicine is made from the ethanol extract of flowers in *Abelmoschus manihot*. A multicenter randomized double-blind parallel controlled clinical trial is designed to evaluate the effect of combined Huangkui capsule and irbesartan treatment on DKD, and the primary outcomes are changed values of albumin-to-creatinine ratio from baseline after treatment. Combined Huangkui capsule and irbesartan treatment exhibits beneficial effect on alleviating albuminuria in patients with type 2 diabetes and DKD (Zhao et al., 2022). Additionally, single Huangkui capsule therapy and combined Huangkui capsule and losartan therapy exert favorable therapeutic effect against primary glomerular disease in a prospective, multicenter randomized controlled clinical trial (Zhang et al., 2014). A multicenter double-blinded randomized placebo-controlled trial shows that accompanying by conventional ARB or ACEI treatment, Tangshen Formula treatment for continuous 24 weeks exhibits obviously beneficial efficacy compared with placebo on decreasing proteinuria and improving eGFR in DKD patients with macroalbuminuria (Li et al., 2015). In this study, primary outcomes are urinary protein level, measured by urinary albumin excretion rate (UAER) for participants with microalbuminuria, 24-h urinary protein for participants with macroalbuminuria. Except for UAER, Tangshen Formula treatment exhibits favorable effects on other primary outcomes. Further investigation indicates that urinary liver-type fatty acid binding protein is identified as the biomarker for the severity of DKD and the effects of Tangshen Formula against DKD (Yang et al., 2016a). The on-going RCT of Tangshen Formula aims to investigate its effectiveness and safety on treating type 2 DKD patients with macroalbuminuria (Yan et al., 2016). The meta-analysis of RCTs shows the satisfied efficacy of combined *Tripterygium wilfordii* Hook, tripterygium glycosides, Ophiocordyceps sinensis, or Jinshuibao with conventional ACEI or ARB treatment against DKD (Luo et al., 2015; Lu et al., 2018; Zhang et al., 2019b; Ren et al., 2019; Wu et al., 2020; Yang et al., 2020).

Notably, several RCT protocols have been designed to provide solid evidences for combined Chinese herbal medicine and conventional ARB or ACEI therapy against DKD. An assessor-blind, parallel, pragmatic randomized controlled clinical trial registered in Hong Kong has been engaged to evaluate the effectiveness of add-on astragalus in clinics. This trial plans to enroll 181 patients with type 2 diabetes, stage 2-3 CKD and macroalbuminuria who receive 48 weeks of add-on astragalus or standard medical care (Chan et al., 2021). A double-blind, placebo-controlled, randomized trial is designed to explore the efficacy and safety of combined Liuwei Dihuang pills with conventional metformin and ARB therapy against DKD for 4 weeks treatment and 12 weeks follow-up, and 24 h urinary protein levels from the baseline to the end of the treatment phase is the primary outcome (Liao et al., 2020). A prospective, single-center RCT aims to investigate the efficacy and safety of combined *Tripterygium* glycosides and ARB therapy for DKD treatment. The primary endpoint is 24 h proteinuria decreased level after treatment for 48 weeks (Lengnan et al., 2020).

## Conclusion and perspectives

DKD is the leading cause of CKD and ESRD worldwide. Although the drug development of RAAS and SGLT2 inhibitors has been evolved, a large proportion of DKD patients still needs dialysis and renal transplantation. The beneficial efficacy of Chinese herbal medicine in clinical application attracts a lot attention as an alternative therapy. DKD progression is normally considered irreversible, while Chinese herbal medicine gives us hope. Reliable evidences from RCTs show that Tangshen Formula and *T. wilfordii* Hook extract significantly reduce proteinuria and elevate eGFR compared with ARB or ACEI (Ge et al., 2013; Li et al., 2015; Liu et al., 2021a). Chinese herbal medicine normally targets multiple and synergetic targets to alleviate DKD due to multiple active compounds, including the improvement of metabolism regulation, the inhibition of oxidative stress and inflammation, the modulation of mitochondrial dysfunction, and the regulation of gut microbiota and related metabolism. Notably, we notice that many Chinese herbal medicines synergistically target multiple key factors and pathways to ameliorate DKD, including icariin, catalpol, *C. tinctoria*, salidroside, and 4-O-methylhonokiol (Table 2). These promising candidates highlight the advantage that synergistically targeting multiple key factors and pathways is the important strategy to facilitate drug development for DKD treatment. These also highlight the importance and urgency to discover and identify the novel therapeutic target. Another advantage of Chinese herbal medicine is its clinical experience for thousands of years in east Asia. Chinese herbal medicine has been still used for prevent and treat DKD today, and many high-quality clinical evidences confirm the efficacy of combined Chinese herbal medicine and conventional western medicine, such as Tangshen Formula, Xiaokeyinshui formula, and *T. wilfordii*, which provide an alternative strategy for DKD treatment.

However, some limitations hinder the recognition and extensive use of Chinese herbal medicine beyond east Asia. One is that the lack of high-quality evidence to identify therapeutic mechanism of Chinese herbal medicine. Most research reported the modulation of Chinese herbal medicine on common mechanism rather than specific and targeted mechanism *via* high throughput analysis. Considerable work needs to be done to identify targeted mechanism of Chinese herbal medicine on DKD treatment. The multiple active compounds of Chinese herbal medicine contribute to the beneficial efficacy to alleviate DKD, but also results in the difficulty to control the quality of Chinese herbal medicine. The identification of active compounds and establishment of corresponding quality control system are necessary to ensure the efficacy and safety of Chinese herbal medicine. Additionally, strict and standardized toxicological research is essential to ensure the acceptable side effect. The proper dosage and duration of Chinese herbal medicine should be investigated in preclinical and clinical trial to ensure favorable efficacy. The lack of high-quality evidence from clinical medicine significantly hinders the extensive use of Chinese herbal medicine, and more efforts are needed to solve this problem. Fortunately, RCTs and mechanism studies of some Chinese herbal medicines have been completed and some is ongoing. For example, the clinical efficacy of Tangshen Formula has been investigated by RCTs, and Tangshen Formula exerts better efficacy in reducing albuminuria and elevating eGFR (Li et al., 2015). Meanwhile, mechanism studies show that Tangshen Formula synergistically targets multiple key factors or pathways to ameliorate DKD *via* modulating pyroptosis, enhancing renal cholesterol efflux, reshaping gut microbiota, and suppressing inflammation (Liu et al., 2018; Li et al., 2020; Zhao et al., 2020). Tangshen Formula sets up a good example that provides reliable evidences for clinical trial and therapeutic mechanism.

Given that many Chinese herbal medicines have yet to be investigated using a modern pharmacological approach, we anticipate many of them could be completed in the future. Chinese herbal medicine has its own advantages in treating DKD, including multiple therapeutic targets and rich clinical experience. New guidelines concerning Chinese herbal medicine against DKD are needed to assure safety and efficacy to amplify its application in treating DKD worldwide.
